# Downregulation of miRNA-214 in cancer-associated fibroblasts contributes to migration and invasion of gastric cancer cells through targeting FGF9 and inducing EMT

**DOI:** 10.1186/s13046-018-0995-9

**Published:** 2019-01-15

**Authors:** Ruifen Wang, Yeqi Sun, Wenwei Yu, Yu Yan, Meng Qiao, Ruiqi Jiang, Wenbin Guan, Lifeng Wang

**Affiliations:** Department of Pathology, School of Medicine, Xinhua Hospital, Shanghai Jiao Tong University, Shanghai, 200092 China

**Keywords:** Cancer-associated fibroblasts, Epithelial–mesenchymal transition, FGF9, Gastric cancer, microRNA-214

## Abstract

**Background:**

Cancer-associated fibroblasts (CAFs), one of the principal constituents of the tumor microenvironment, have a pivotal role in tumor progression. Dysregulation of microRNAs (miRNAs) in CAFs contributes to the tumor-promoting ability of CAFs. However, the mechanism underlying the involvement of miRNAs in CAFs of gastric cancer (GC) is not fully understood. This study aimed to explore the effects of miRNA-214 in CAFs on GC migration and invasion.

**Methods:**

The primary CAFs and corresponding normal fibroblasts (NFs) were isolated. Cell counting kit-8, EdU cell proliferation staining and Transwell assays were used to determine the role of miRNA-214 in GC progression. Real-time polymerase chain reaction, Western blot analysis, and dual-luciferase reporter assay were performed to verify the target genes of miRNA-214. Immunofluorescence and Western blot analysis were applied to detect the expression of epithelial–mesenchymal transition (EMT) markers. Immunohistochemistry and in situ hybridization were implemented to analyze the fibroblast growth factor 9 (FGF9) and miRNA-214 expression in human GC tissues, respectively. Finally, to assess its prognostic relevance, Kaplan–Meier survival analysis was conducted.

**Results:**

MiRNA-214 was significantly downregulated in CAFs of GC compared with NFs. The upregulation of miRNA-214 in CAFs inhibited GC cell migration and invasion in vitro but failed to affect proliferation. Moreover, GC cells cultured with conditioned medium from CAFs transfected with miR-214 mimic showed increased expression of E-cadherin and decreased expression of Vimentin, N-cadherin and Snail, indicating the suppression of EMT of GC cells. Furthermore, FGF9 was proved to be a direct target gene of miR-214. The expression of FGF9 was higher in CAFs than that in tumor cells not only in primary tumor but also in lymph node metastatic sites (30.0% vs 11.9%, *P* < 0.01 and 32.1% vs 12.3%, *P* < 0.01, respectively). Abnormal expression of FGF9 in CAFs of lymph node metastatic sites was significantly associated with poor prognosis in patients with GC (*P* < 0.05).

**Conclusions:**

This study showed that miR-214 inhibited the tumor-promoting effect of CAFs on GC through targeting FGF9 in CAFs and regulating the EMT process in GC cells, suggesting miRNA-214/FGF9 in CAFs as a potential target for therapeutic approaches in GC.

**Electronic supplementary material:**

The online version of this article (10.1186/s13046-018-0995-9) contains supplementary material, which is available to authorized users.

## Background

Gastric cancer (GC) is globally one of the most common cancers with high mortalities, especially in Asia. According to the cancer statistics in China in 2015, GC is the second most common cancer and the second leading cause of cancer death [1]. Although chemotherapy and targeted therapy (trastuzumab) benefit a part of patients, tumor invasion and metastasis remain major obstacles in treatment and prognosis. The “seed and soil” theory of tumor metastasis unveiled the limitation of designing treatment strategies that focus on cancer cells per se, thus, more attention should be given to the crosstalk between tumor cells and tumor microenvironment (TME). Over the years, it has been increasingly acknowledged that tumor progression and metastasis is instigated by the bidirectional communication between tumor cells and TME, rather than cancer cells alone [2]. Unlike tumor cells, stromal cells within the TME are genetically stable, making them a more attractive therapeutic target for cancer therapy. Cancer-associated fibroblasts (CAFs), one of the most important stromal cell types in TME, have been considered as co-conspirators of tumor progression [3, 4]. Genomic meta-analysis and histopathological evaluation identified that high stromal content is associated with poor prognosis in GC [5]. Accumulating evidence suggests that CAFs contribute to GC development and are potential therapeutic targets for GC [6, 7]. In previous studies, CAFs separated from GC tissues had an essential role in the migration and invasion of GC cells [8, 9]. Nevertheless, the mechanisms underlying the tumor-promoting effect of CAFs on GC cells remain obscure.

Somatic genetic alterations in CAFs are exceedingly rare and unlikely to be responsible for stable cancer-promoting properties of CAFs as reported by Qiu [10]. Therefore, epigenetic changes possibly led to the tumor-promoting phenotype of CAFs. In recent years, microRNAs (miRNAs) regulation is considered to be one of the most common epigenetic modifications. MiRNAs are small, noncoding RNAs, which function as oncogenes or anti-oncogenes by suppressing the translation of their target mRNAs. Emerging literature have elucidated that dysregulation of miRNAs is crucial for tumor-stromal interactions between cancer cells and CAFs [11, 12]. However, it is still not very clear about the exact function of miRNAs in CAFs of GC. The result of this study exhibited that downregulation of miRNA-214 in CAFs caused the migration and invasion of GC cells via activating epithelial–mesenchymal transition (EMT) process. It was further observed that fibroblast growth factor 9 (FGF9) as a direct target gene of miRNA-214 was overexpressed in CAFs and associated with poor prognosis of patients with GC.

## Methods

### Cell lines and isolation of primary fibroblasts

GC cell lines MGC-803 and SGC-7901, and 293 T cells were obtained from Shanghai Institutes for Biological Sciences and cultured in RPMI 1640 or DMEM medium supplemented with 10% fetal bovine serum at 37 °C. The primary NF and CAF populations were isolated from the normal zone (more than 5 cm far from the tumor zone) and tumor zone (tissue within the tumor boundary) of human gastric tissues of the same patient, as described in a previous study [9]. None of the patients had received preoperative radiotherapy or chemotherapy. The NFs or CAFs were cultured in DMEM medium supplemented with 10% fetal bovine serum at 37 °C. The cells were incubated until they were 70–90% confluent, washed with phosphate-buffered saline (PBS) three times, and finally cultured in serum-free media for another 48 h to prepare conditioned medium (CM). The CM was collected and centrifuged for 10 min at 3000 rpm to remove cell debris. CM was stored at − 80 °C until use. Three representative pairs of NFs and CAFs were used in this study. All fibroblasts used for in vitro study were 3–10 passages.

### Immunocytochemistry and immunofluorescence

The cells were fixed in 4% paraformaldehyde for 20 min and permeabilized with 0.1% Triton X-100 for 30 min. Primary antibodies including fibroblast activation protein (FAP, R&D Systems, MN, USA), Vimentin (Dako, CA, USA), Cytokeratin (Dako), and E-cad (Dako) were added to each chamber, and the slides were incubated overnight at 4 °C. Next, the cells were incubated with a horseradish peroxidase (HRP)-conjugated secondary antibody (for immunocytochemistry) or FITC-conjugated secondary antibody (for immunofluorescence) for 1 h at room temperature. The cells were then stained with diaminobenzidine (DAB) or DAPI for 5 min.

### Cell transfection

Oligonucleotides for hsa-miR-214 mimics (miR-214) and negative control (miR-NC) were purchased from GenePharma (Shanghai, China). The sequence of hsa-miR-214 mimics and negative control are shown in Additional file 1 Table S1. Briefly, the cells were seeded at 70–90% confluence the day before transfection. Cells were transfected with oligonucleotides using lipofectamine 2000 (Invitrogen, CA, USA) at a final concentration of 100 nM according to the manufacturer’s protocol. The transfection efficiency of miR-214 was verified after incubated for 48 h, and the cells were subsequently used for real-time polymerase chain reaction (PCR) and Western blot analysis.

### Cellular RNA extraction and quantitative real-time PCR

Total RNA was isolated from cells with TRIzol reagent (TaKaRa, Dalian, China) according to the manufacturer’s protocol. Total RNA (500 ng) was reverse transcribed with the PrimeScript RT reagent kit (TaKaRa) in a final volume of 10 μL. Quantitative real-time PCR (qRT-PCR) was performed using the SYBR Premix Ex Taq (TaKaRa) and analyzed with the ABI 7500 real-time PCR system. The expression levels of miRNAs were measured using a Mir-X miRNA First Strand Synthesis Kit (TaKaRa) according to the manufacturer’s protocol. U6 and GAPDH were used as internal controls for miRNAs and mRNAs, respectively. Each sample was analyzed in triplicate. The primers for qRT-PCR are listed in Additional file 1: Table S2.

### Western blot analysis

The cells were collected and lysed with cell lysis buffer for the Western blot analysis. The proteins in the lysates (20 μg per lane) were separated on 8% or 10% sodium dodecyl sulfate–polyacrylamide gel electrophoresis and transferred onto polyvinylidene fluoride membranes. After blocking with 5% nonfat milk in Tris-buffered saline with Tween 20 (TBST), the primary antibodies for FAP (R&D), FGF9 (R&D), E-cad (Cell Signaling Technology, MA, USA), N-cad (Cell Signaling Technology), Snail (Cell Signaling Technology), and GAPDH (Beyotime, Shanghai, China) were used overnight at 4 °C. The membranes were then washed with TBST three times and incubated with appropriate secondary antibodies. The protein bands were visualized using the enhanced chemiluminescence reagents (Millipore, MA, USA).

### Cell proliferation assay

The cell proliferation assay was performed using a Cell Counting Kit-8 (CCK-8, Dojindo, MD, USA) according to the manufacturer’s protocol. MGC-803 or SGC-7901 cells (2 × 10^3^ cells/well) were seeded in 96-well plates and cultured in CAFs ^miR-214^-CM or CAFs^NC^-CM. The optical density at 450 nm wavelength (OD450) was measured every 24 h for 5 days. EdU cell proliferation staining was performed using an EdU kit (BeyoClick™ EdU Cell Proliferation Kit with Alexa Fluor 488, Beyotime, China). Briefly, MGC-803 or SGC-7901 cells (2 × 10^4^ cells/well) were seeded in 24-well plates and cultured in CAFs ^miR-214^-CM or CAFs^NC^-CM for 72 h or 96 h, respectively. Subsequently, cells were incubated with EdU for 3 h, fixed with 4% paraformaldehyde for 15 min, and permeated with 0.3% Triton X-100 for another 15 min. The cells were incubated with the Click Reaction Mixture for 30 min at room temperature in a dark place and then incubated with Hoechst 33342 for 10 min.

### Cell migration and invasion assays

The migration and invasion abilities of GC cells were assayed using Transwell inserts and Matrigel-coated Transwell (Corning, MA, USA). In brief, the CM collected from CAFs or NFs was added into the lower chamber of a 24-well plate. MGC-803 and SGC-7901 cells, with a density of 2 × 10^4^ cells in 200 μL of serum-free medium, were added into the Transwell chambers with the Matrigel member covered or uncovered. Then, the cell plate was incubated in a humidified atmosphere (37 °C and 5% CO_2_). After culturing for 24 h (migration assays) or 48 h (invasion assay), the cells on the upper surface of the membrane were completely removed using a cotton swab. Then, the cells were fixed in 4% paraformaldehyde and stained with crystal violet solution for 15–20 min. The migrating or invading cells were counted under a microscope and photographed.

### Luciferase reporter assay

The 293 T cells were cultured in a 24-well plate. The next day, the cells were transfected with a luciferase construct containing wild-type or mutated binding site vectors of FGF9 3′-untranslated region (3’UTR) and co-transfected with pre-miR-214 or negative control plasmids. The luciferase vectors were constructed by GeneChem Company (Shanghai). The sequence of pre-miR-214 was as follows: 5’-GGCCTGGCTGGACAGAGTTGTCATGTGTCTGCCTGTCTACACTTGCTGTGCAGAACATCCGCTCACCTGTACAGCAGGCACAGACAGGCAGTCACATGACAACCCAGCCT-3′. After 48 h of transfection, firefly and Renilla luciferase activities were measured using the dual-luciferase kit (Promega, WI, USA) according to the manufacturer’s protocol.

### Patients and tissue sample collection

The 160 patients with GC and corresponding lymph nodes were assessed from 2011 to 2015 at the Department of Pathology, Xinhua Hospital, School of Medicine, Shanghai Jiao Tong University. These tissues were formalin-fixed and paraffin-embedded (FFPE). Primary tumor (*n* = 160) and lymph node metastatic sites (*n* = 106) were used for tissue microarrays. The clinicopathological features and follow-up data of patients were collected from hospital information and follow-up.

### Immunohistochemistry

The slides were deparaffinized and rehydrated using routine methods. Briefly, the slides were washed with PBS. The endogenous peroxidase activity was blocked with 3% H_2_O_2_ for 10 min, and then the slides were boiled in EDTA buffer (pH = 9) for 20 min to retrieve antigenicity. After incubation with bovine serum albumin for 20 min at room temperature, the slides were incubated with primary antibody FGF9 (1:150, R&D systems) overnight at 4 °C and then with HRP-conjugated secondary antibody at room temperature for 30 min. DAB was used for staining. The expression of FGF9 was calculated using the proportion score and intensity score by two experienced pathologists. The proportion score of positive cells was as follows: 0, negative; 1, < 25% positive cells; 2, 26–50% positive cells; and 3, > 50% positive cells. The staining intensity score was as follows: 0, absence; 1, weak expression; 2, moderate expression; and 3, strong expression. The total scores from 0 to 4 were regarded as a low level of expression, and scores from 5 to 6 were classified as a high level of expression.

### In situ hybridization (ISH)

To detect the miRNA214 in gastric cancer, FFPE tissue microarray was used in situ hybridization. The digoxigenin-labled miR-214 detection probe was synthesized by the Boster Biological Technology co.ltd (China), and the corresponding ISH Detection Kit I (MK1030), DAB kit (AR1022) were also purchased from this company. The procedures indicated as follows simply. According to the instruction, tissue sections were incubated with miR-214 probe at 38 °C overnight, treated 1 h at 37 °C by biotinylated mouse anti-digoxigenin on the next day and stained 20–30 min by the DAB.

### Statistical analysis

Data were summarized as mean ± standard deviation from three independent experiments. The statistical data were analyzed by the *χ*^2^-test and Student *t* test using SPSS19.0 software. The Kaplan–Meier analysis and log-rank test were performed for the survival analysis using Stata15.0 software. A two-tailed *P* value <0.05 was considered statistically significant.

## Results

### Characterization of primary cultured NFs and CAFs

**Fig. 1:**
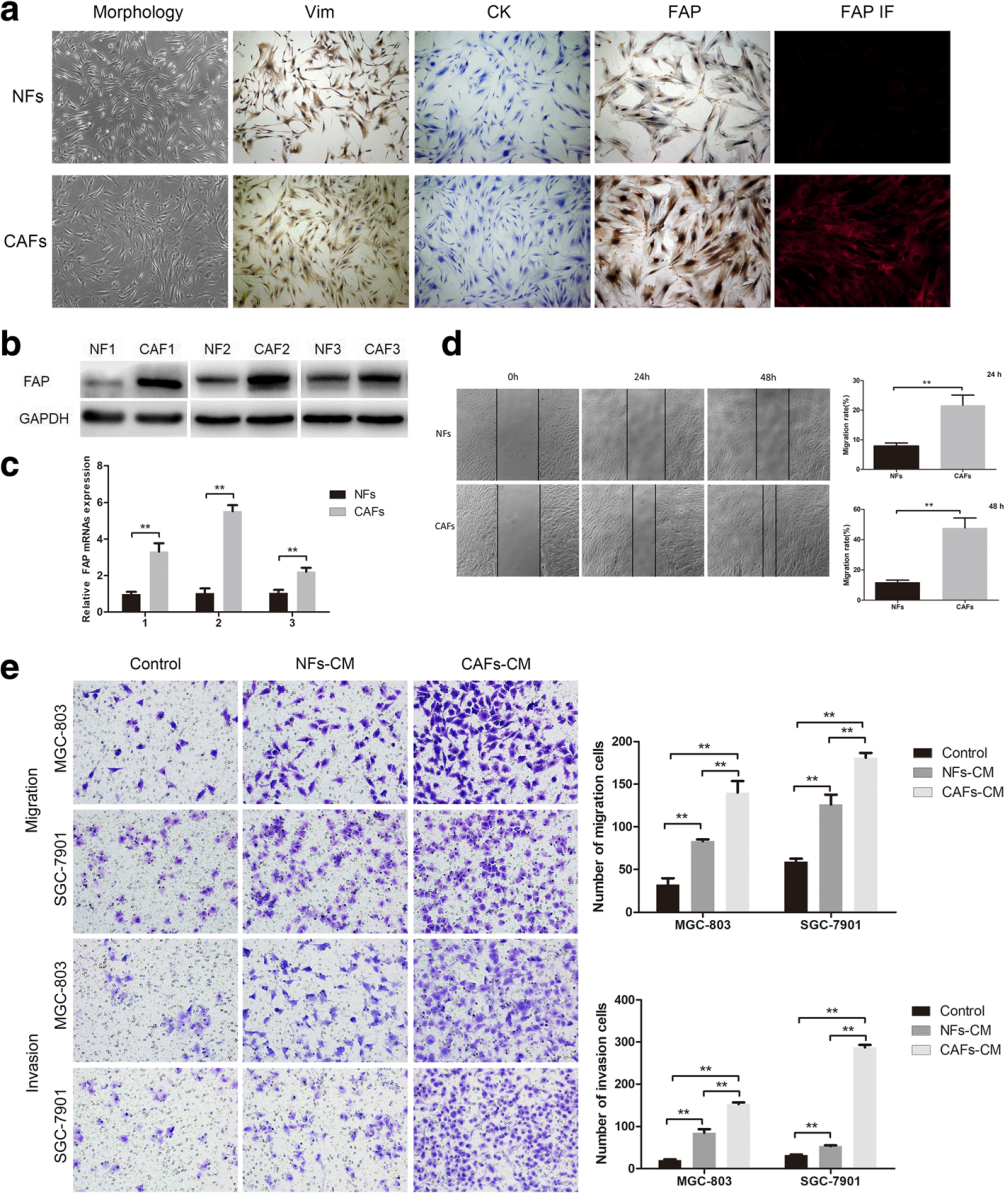
Characterization of primary cultured NFs and CAFs and their effects on migration and invasive ability of GC cells. **a** The morphology of gastric NFs and CAFs (left). Immunocytochemical staining showed the expression of Vimentin, Cytokeratin, and FAP in NFs and CAFs (middle), and immunofluorescence staining for FAP (right). **b** Western blot analysis of FAP expression in three paired NFs and CAFs. **c** The mRNA expression levels of FAP in three paired NFs and CAFs. **d** The migration ability of CAFs itself was stronger than that of paired NFs at 48 h and 72 h. **e** CAFs-CM significantly promoted the migration and invasive ability of MGC-803 and SGC-7901 cells than NFs-CM. (*P < 0.05, **P < 0.01)

The NF and CAF populations were successfully isolated from the normal and tumor zones of human GC tissue of the same patient, respectively. Both NFs and CAFs showed a long spindle-like morphology, but CAFs were slightly plump than NFs (Fig. 1a). The expression of fibroblast biomarker in these primary cultured cells was examined to test the purity of NFs and CAFs. As shown in Fig. 1a, primary cultured fibroblast populations (NFs and CAFs) were strongly positive for mesenchymal marker (Vimentin, Vim), but negative for epithelial marker (Cytokeratin, CK). Both the immunocytochemistry and immunofluorescence staining showed that FAP (a special CAF biomarker) was overexpressed in CAFs compared with NFs. Western blot analysis and qRT-PCR assay further confirmed that the protein and mRNA expression levels of FAP significantly increased in CAFs compared with NFs (Fig. 1b and c). Altogether, these data indicated the successful isolation of gastric NFs and CAFs with high purity. Moreover, the wound healing assay revealed that the migration ability of CAFs itself was stronger than that of NFs at 48 h and 72 h (Fig. 1d).

### CAFs induced migration and invasion of GC cells

MGC-803 and SGC-7901 cells were treated with CAF conditioned medium (CAF-CM), NF conditioned medium (NF-CM) or control medium (DMEM). The results showed (Fig. 1e) that CAF-CM and NF-CM could remarkably increase the number of migrated and invaded GC cells compared with control. In contrast to NF-CM, CAF-CM greatly increased the migration and invasion abilities of GC cells. These results strongly supported that CAFs could facilitate the migration and invasion of GC cells than NFs in vitro.

### miR-214 was downregulated in gastric CAFs and critical for the tumor-promoting ability of CAFs

**Fig. 2:**
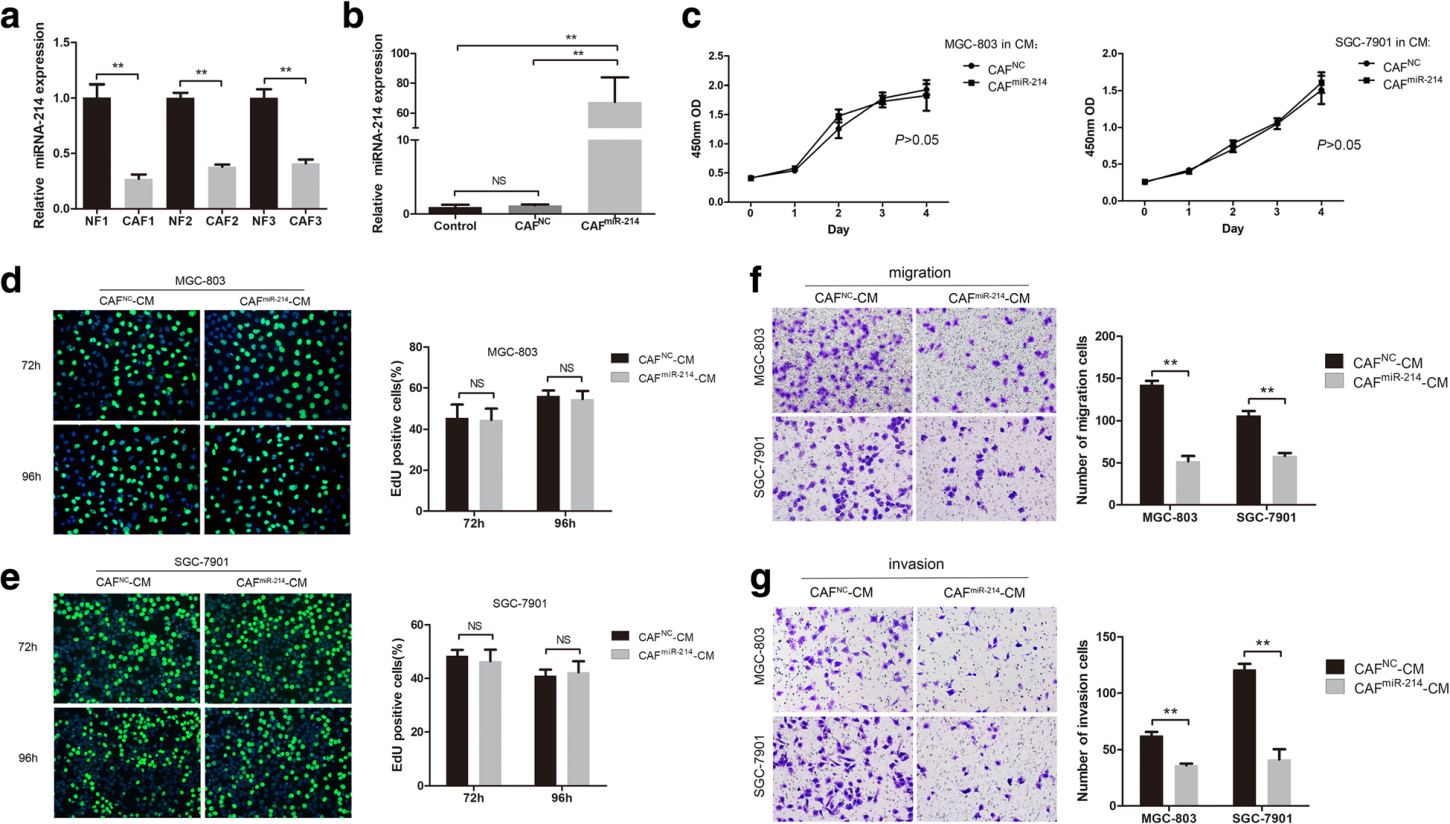
MiR-214 suppressed the migration and invasion but not proliferation ability of GC cells. **a** Expression of miR-214 was significantly downregulated in CAFs than in paired NFs. **b** The ectopic expression of miR-214 in CAFs transfected with miR-214 mimics was confirmed by qRT-PCR. **c**, **d** and **e** The CCK8 and EdU assays showed that CAFs^miR-214^-CM did not influence the proliferation of MGC-803 and SGC-7901 cells compared with CAFs^NC^-CM (no significant difference, NS, *P* > 0.05). **f** and **g** CAFs^miR-214^-CM markedly inhibited the migration and invasion of MGC-803 and SGC-7901 cells compared with CAFs^NC^-CM (**P < 0.01)

In preliminary work, due to the uncertain role of dysregulated miRNAs in gastric CAFs and NFs, we searched published datasets in PubMed records and unfolded dysregulation of 18 miRNAs in CAFs (miRNA-31, miRNA-155, miRNA-483-3p, miRNA-26a, miRNA-148a, miRNA-93, let-7 g, miRNA-106b, miRNA-424, miRNA-149, miRNA-199a, miRNA-21, miRNA-34b, miRNA-101, miRNA-301a, miRNA-145, miRNA-143, and miRNA-214) from different types of cancer [13–18]. The expression of these 18 miRNAs was examined in CAFs and NFs using the qRT-PCR assay to identify CAF-specific dysregulated miRNAs in GC. Among them, the most downregulated miRNA in CAFs was miRNA-214 [19]. Further, in this validation study, miRNA-214 expression was verified significantly downregulated in CAFs than in paired NFs (Fig. 2a). Then, CAFs were transfected with miRNA-214 mimics or negative control (CAFs^miR-214^ or CAFs^NC^) to explore whether miRNA-214 was involved in the tumor-promoting ability of fibroblasts. And the ectopic expression efficiency was confirmed by qRT-PCR (Fig. 2b). After that, the effects of conditioned medium from CAFs^miR-214^ or CAFs^NC^ (CAFs^miR-214^-CM or CAFs^NC^-CM) on the proliferation, migration, and invasion of GC cells were investigated. The CCK-8 and EdU results showed that the proliferation ability of GC cells cultured in CAFs^miR-214^-CM was not changed when compared with that in CAFs^NC^-CM (Fig. 2c, d and e),whereas, the former CM markedly inhibited the migration and invasion of GC cells (Fig. 2f and g). Taken together, these results indicated that tumor suppressor miRNA-214 in CAFs decreases the migration and invasion but not proliferation of GC cells.

### CAFs enhanced epithelial–mesenchymal transition of GC cells in a miR-214-dependent manner

**Fig. 3:**
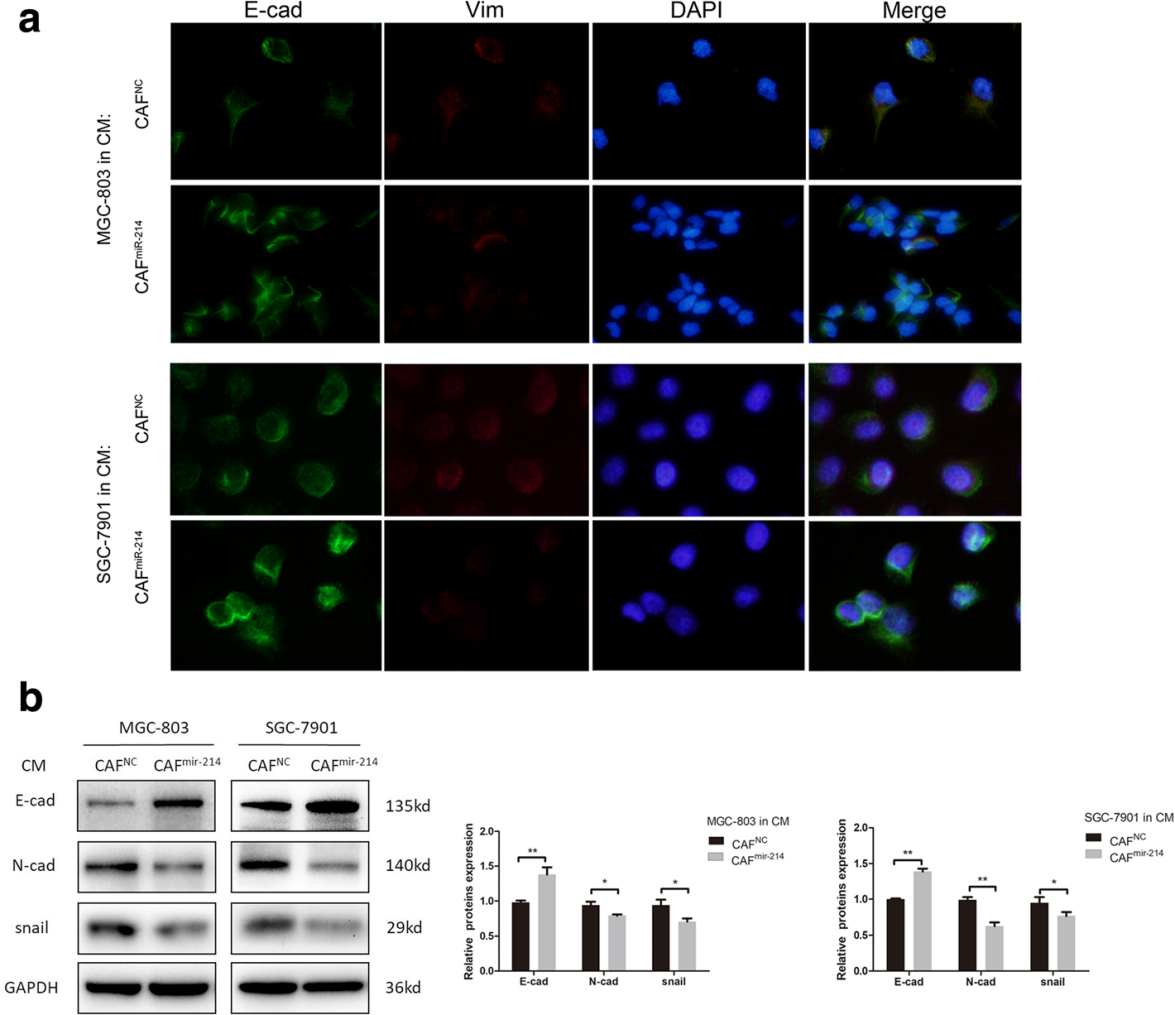
CAFs enhanced epithelial–mesenchymal transition of GC cells in a miR-214-dependent manner. **a** Immunofluorescence detection showed that CAFs^miR-214^-CM enhanced the expression of E-cadherin and suppressed the expression of Vimentin of GC cells. **b** The Western blot analysis showed that CAFs^miR-214^-CM significantly increased E-cadherin expression and decreased N-cadherin and Snail expression in GC cells (*P < 0.05, **P < 0.01)

There is growing evidence that CAFs can stimulate a key process in tumor invasion and metastasis, namely epithelial–mesenchymal transition (EMT). Hence, this study investigated whether miR-214 expression in CAFs could influence the EMT of GC cells. Using immunofluorescence and Western blot assays, the expression of the epithelial cell marker E-cadherin, the mesenchymal cell markers Vimentin and N-cadherin, and the transcription factor Snail were analyzed in GC cells treated with CAFs^miR-214^-CM or CAFs^NC^-CM. Immunofluorescence detection showed that the CM from CAFs^miR-214^ instead of CAFs^NC^ could enhance E-cadherin expression and suppress Vimentin expression (Fig. 3a). These findings were subsequent confirmed by Western blot analysis. It is likely that, CAFs enhanced EMT in a miR-214-dependent manner as indicated by the fact that CAFs^miR-214^-CM restraining EMT of GC cells by increasing E-cadherin and decreasing N-cadherin and Snail expression (Fig. 3b).

### MiR-214 inhibited the tumor-promoting ability of CAFs by targeting FGF9

In light of the reported methods [20–23], the bioinformatics databases (TargetScan, miRDB, and microRNA.org) were searched to predict potential target genes of miR-214 so as to explore its possible mechanisms of action. A number of growth factors, inflammatory factors, and secreted factors (e.g. FGF9, FGF14, FGF11, FGF1, FGF7, FGF10, IL3, CCL

**Fig. 4:**
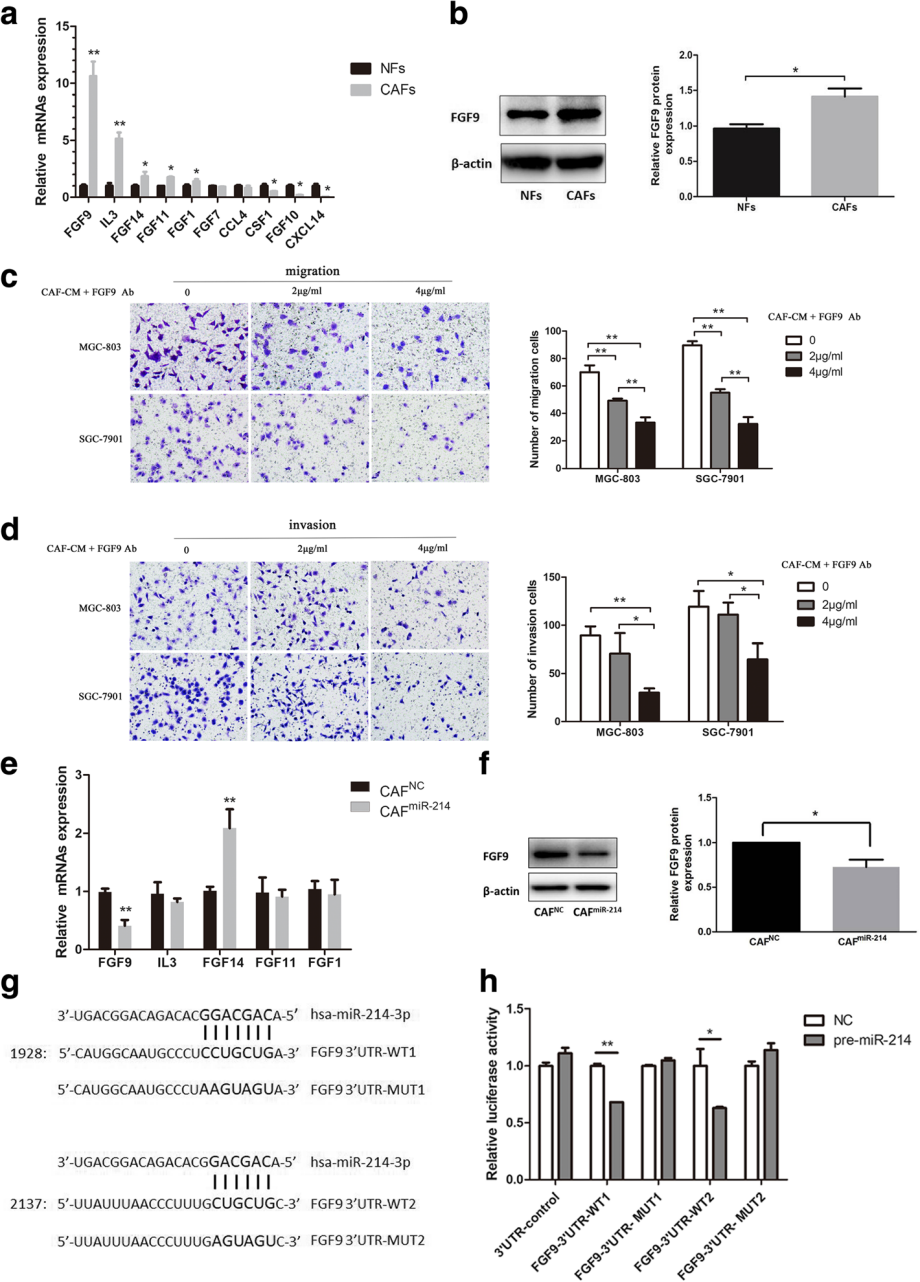
MiR-214 inhibited the tumor-promoting ability of CAFs by directly targeting FGF9. **a** Ten predicted genes as potential targets of miR-214 were analyzed by qRT-PCR. Five of them were overexpressed in CAFs compared to NFs, in which FGF9 represented the most prominent one. **b** The protein expression levels of FGF9 in NFs and CAFs. **c** and **d** The migration and invasion abilities of cultured GC cells were suppressed after adding FGF9 neutralizing antibody into CAF-CM. **e** Among five upregulated genes as mentioned above, only FGF9 mRNA expression was reduced in CAF^miR-214^ compared to CAF^NC^. **f** FGF9 protein expression in CAFs was also suppressed in CAF^miR-214^ compared to CAF^NC^. **g** Two predicted binding sites between miR-214 and FGF9 3’UTR. **h** The relative luciferase activity was significantly suppressed in cells co-transfected with wild-type binding site vectors of FGF9 3’UTR in the presence of pre-miR-214 (*P < 0.05, **P < 0.01)

4, CSF1, and CXCL14) were collected and then surveyed in CAFs and NFs. The expression of five of the genes was elevated in CAFs compared to NFs, in which FGF9 represented the most prominent one (Fig. 4a, *P* < 0.01). FGF9 expression also radically increased in CAFs than that in NFs at protein level (Fig. 4b, *P* < 0.01). Moreover, FGF9 neutralizing antibody was added into CAF-CM to treat GC cells so as to further clarify the effect of FGF9 in CAFs on tumor cell migration and invasion. The results showed that FGF9-neutralizing antibody could inhibit the migration and invasion of GC cells (Fig. 4c and d). Furthermore, to ascertain whether FGF9 was a target gene of miR-214, the miR-214 mimics were transfected into CAFs to upregulate the miR-214 level, followed by evaluation of five genes above. The results displayed that FGF9 mRNA and protein expression levels were notably decreased by miR-214 mimics in CAFs (Fig. 4e and f; *P* < 0.01, *P* < 0.05, respectively). A dual-luciferase reporter assay was performed to further measure whether FGF9 was a direct target gene of miR-214. Two potentially predicted binding sites were found between FGF9 3’UTR and miR-214 (Fig. 4g). The relative luciferase activity was appreciably suppressed in cells co-transfected with wild-type binding site vectors of FGF9 3’UTR in the presence of pre-miR-214. This inhibitory effect was noticed at both predicted binding sites. However, cells co-transfected with the mutated binding site vectors of FGF9 3’UTR could not decrease the luciferase activity (Fig. 4h). The results revealed that FGF9 was a direct target of miR-214. In all, these results indicated that FGF9 offered considerable benefit for the tumor-promoting ability of CAFs, and miR-214 inhibited the tumor-promoting ability of CAFs possibly by directly reducing FGF9 expression.

### High FGF9 expression in CAFs of lymph nodes was associated with poor prognosis

**Fig. 5:**
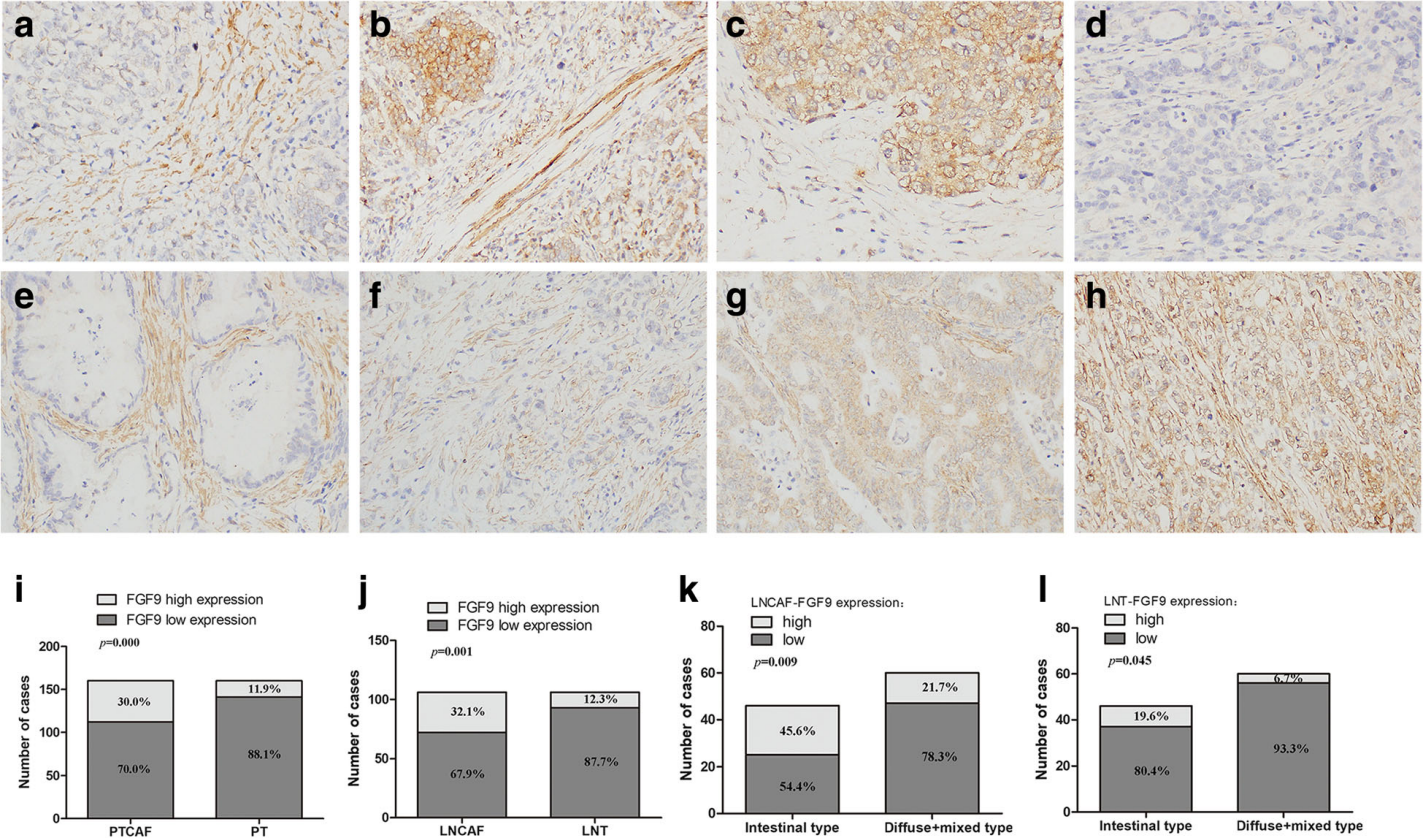
FGF9 expression in primary tumor (**a**–**d**) and lymph node metastatic sites (**e**–**h**) of GC. **a** The high expression of FGF9 in CAFs, but low expression in tumor cells. **b** CAFs and tumor cells were both positive for FGF9. **c** The high expression of FGF9 in tumor cells, but low staining in CAFs. **d** CAFs and tumor cells were both negative for FGF9. **e** and **f** FGF9 staining was high in CAFs but low in tumor cells of both intestinal type (**e**) and diffuse type (**f**). **g** and **h** CAFs and tumor cells were both positive for FGF9 (**g**, intestinal type and **h**, diffuse type). **i** and **j** The proportion of high expressed FGF9 in CAFs and tumor cells of primary tumor (**i**) and lymph node metastatic sites (**j**). The percentage of high expressed FGF9 in LNCAFs (**k**) and LNT (**l**) of intestinal-type and diffused and mixed-type

The FGF9 expression levels in primary GC samples (*n* = 160) and corresponding lymph node metastatic sites (*n* = 106) were evaluated using a tissue microarray for further clarifying the effect of FGF9 on GC progression (Table 1). Among the 129 lymph node metastatic sites, 23 cases were excluded for their too few tumor cells in lymph nodes to perform microarray or in- depth testing. The expression of FGF9 was separately analyzed in primary tumor CAFs (PTCAF-FGF9), primary tumor cell (PT-FGF9), lymph node metastatic site CAFs (LNCAF-FGF9), and tumor cells in lymph node metastatic sites (LNT-FGF9). The immunostaining of FGF9 was cytoplasmic localization with four expression patterns in the primary tumor: (1) the expression was high in CAFs, but low in tumor cells (Fig. 5a). (2) FGF9 expression was positive in both CAFs and tumor cells (Fig. 5b). (3) In a few cases, the expression was high in tumor cells but not in CAFs (Fig. 5c). (4) CAFs and tumor cells were both negative for FGF9 (Fig. 5d). In addition, two features were characterized by FGF9 positive staining in lymph node metastasis sites: First, FGF9 staining was high in CAFs but low in tumor cells and this distribution was more remarkable in intestinal-type than in diffuse-type tumor (Fig. 5e and f). Second, CAFs and tumor cells were both positive for FGF9 (Fig. 5g and h). Moreover, the prevalence of FGF9 high-expression group was significantly increased in CAFs than that in tumor cells not only in primary tumor but also in lymph node metastatic sites (Fig. 5i and j, 30.0% vs 11.9%, *P* < 0.01 and 32.1% vs 12.3%, *P* < 0.01, respectively).

**Table 1:** Correlation between FGF9 expression and clinicopathological parameters of gastric adenocarcinomas

Features	N	PTCAF-FGF9 expression(%)		*p* value	PT-FGF9 expression(%)		*p* value	N	LNCAF-FGF9 expression(%)		*p* value	LNT-FGF9 expression(%)		*p* value
		low	high		low	high			low	high		low	high	
Total	160	112(70.0)	48(30.0)		141(88.1)	19(11.9)		106	72(67.9)	34(32.1)		93(87.7)	13(12.3)	
Age														
< 60	54	42(77.8)	12(22.2)	0.125	53(98.1)	1(1.9)	0.005*	38	30(79.0)	8(21.0)	0.069	36(94.7)	2(5.3)	0.129
≥60	106	70(66.0)	36(34.0)		88(83.0)	18(17.0)		68	42(61.8)	26(38.2)		57(83.8)	11(16.2)	
sex														
male	115	83(72.2)	32(27.8)	0.337	100(87.0)	15(13.0)	0.465	75	50(66.7)	25(33.3)	0.666	63(84.0)	12(16.0)	0.102
female	45	29(64.4)	16(39.6)		41(91.1)	4(8.9)		31	22(71.0)	9(29.0)		30(96.8)	1(3.2)	
tumor size														
< 5	57	45(79.0)	12(21.0)	0.066	53(93.0)	4(7.0)	0.158	38	27(71.1)	11(28.9)	0.606	32(84.2)	6(15.8)	0.408
≥ 5	103	67(65.0)	36(35.0)		88(85.4)	15(14.6)		68	45(66.2)	23(33.8)		61(89.7)	7(10.3)	
Location														
GEJ-Cardia	24	15(62.5)	9(37.5)	0.029*	22(91.7)	2(8.3)	0.392	18	9(50.0)	9(50.0)	0.113	16(88.9)	2(11.1)	1.000
Fundus-Body	41	30(73.2)	11(26.8)		38(92.7)	3(7.3)		28	22(78.6)	6(21.4)		25(89.3)	3(10.7)	
Antrum-Pylorus	87	65(74.7)	22(25.3)		75(86.2)	12(13.8)		57	40(70.2)	17(29.8)		49(86.0)	8(14.0)	
Remnant+ multi-sites	8	2(25.0)	6(75.0)		6(75.0)	2(25.0)		3	1(33.3)	2(66.7)		3(100)	0(0)	
Differentiation														
well-moderate	25	16(64.0)	9(36.0)	0.476	21(84.0)	4(16.0)	0.503	16	7(43.8)	9(56.2)	0.025*	13(81.3)	3(18.7)	0.411
poor	135	96(71.1)	39(28.9)		120(88.9)	15(11.1)		90	65(72.2)	25(27.8)		80(88.9)	10(11.1)	
Lauren type														
intestinal	68	49(72.1)	19 (27.9)	0.625	57(83.8)	11(16.2)	0.148	46	25(54.4)	21(45.6)	0.009*	37(80.4)	9(19.6)	0.045*
diffuse+mixed	92	63(68.5)	29(31.5)		84(91.3)	8(8.7)		60	47(78.3)	13(21.7)		56(93.3)	4(6.7)	
pT stage														
T1 + T2	17	14(82.4)	3(17.6)	0.240	17(100)	0(0)	0.226	12	9(75.0)	3(25.0)	0.577	11(91.7)	1(8.3)	1.000
T3 + T4	143	98(68.5)	45(31.5)		124(86.7)	19(13.3)		94	63(67.0)	31(33.0)		82(87.2)	12(12.8)	
pN stage														
N0	31	18(58.1)	13(41.9)	0.407	28(90.3)	3(9.7)	0.792	/	/	/		/	/	/
N1	35	26(74.3)	9(25.7)		29(82.9)	6(17.1)		20	16(80.0)	4(20.0)	0.175	17(85.0)	3(15.0)	0.599
N2	29	22(75.9)	7(24.1)		26(89.7)	3(10.3)		24	13(54.2)	11(45.8)		20(83.3)	4(16.7)	
N3	65	46(70.8)	19(29.2)		58(89.2)	7(10.8)		62	43(69.4)	19(30.6)		56(90.3)	6(9.7)	
M stage														
M0	157	110(70.1)	47(29.9)	1.000	139(88.5)	18(11.5)	0.317	103	70(68.0)	33(32.0)	1.000	90(87.4)	13(12.6)	1.000
M1	3	2(66.7)	1(33.3)		2(66.7)	1(33.3)		3	2(66.7)	1(33.3)		3(100)	0(0)	
TNM stage														
I + II	63	42(66.7)	21(33.3)	0.458	55(87.3)	8(12.7)	0.795	20	14(70.0)	6(30.0)	0.825	16(80.0)	4(20.0)	0.262
III + IV	97	70(72.2)	27(27.8)		86(88.1)	11(11.9)		86	58(67.4)	28(32.6)		77(89.5)	9(10.5)	

*p < 0.05

**Fig. 6:**
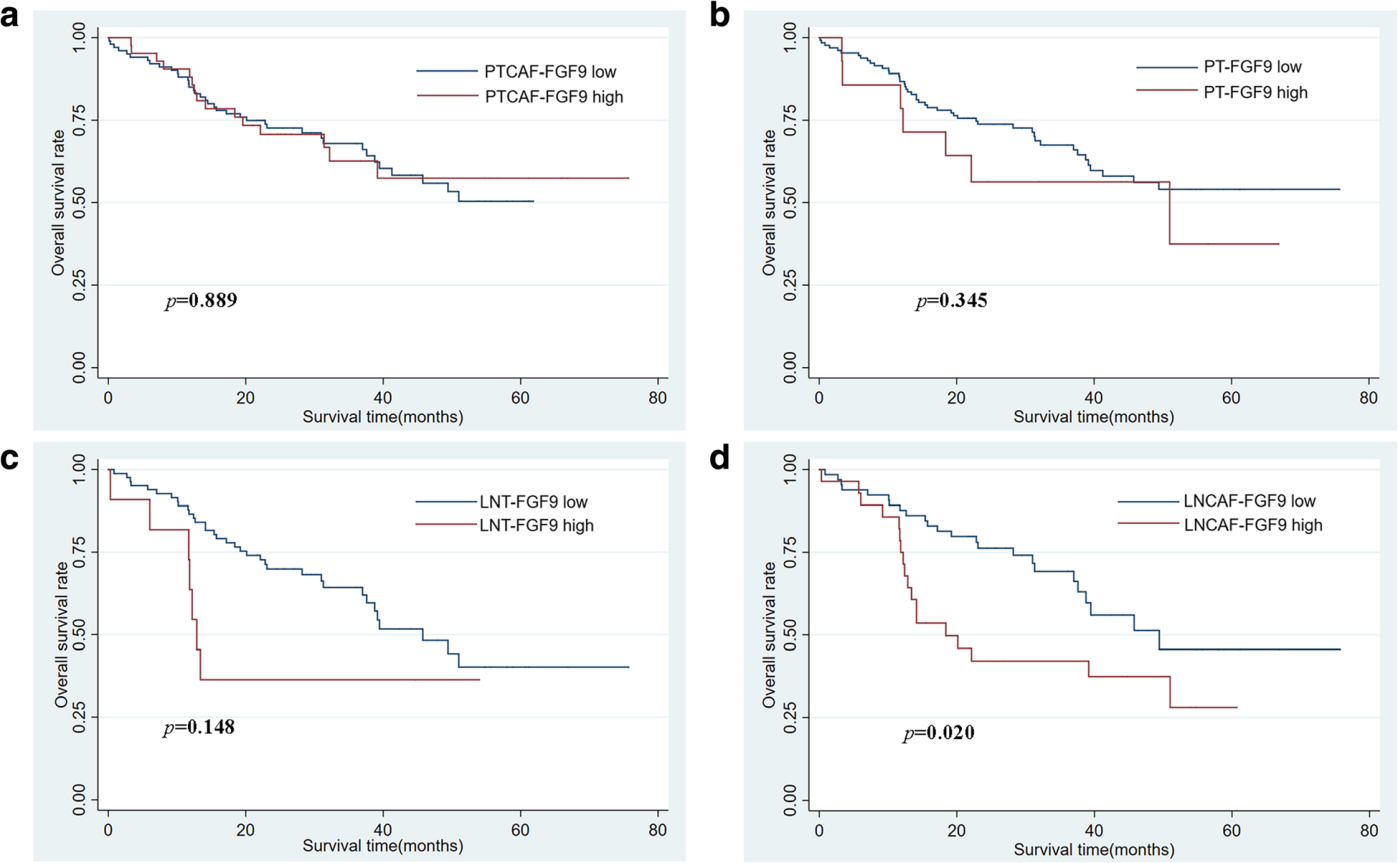
High FGF9 expression in lymph node metastatic sites CAFs was associated with poor prognosis in patients with GC. **a**–**c** The Kaplan–Meier survival analysis revealed that the FGF9 level in primary tumor CAFs, primary tumor cells, and lymph node metastatic sites tumor cells was not associated with prognosis. **d** High FGF9 expression in lymph node metastatic site CAFs was significantly associated with poor prognosis in patients with GC

**Fig. 7:**
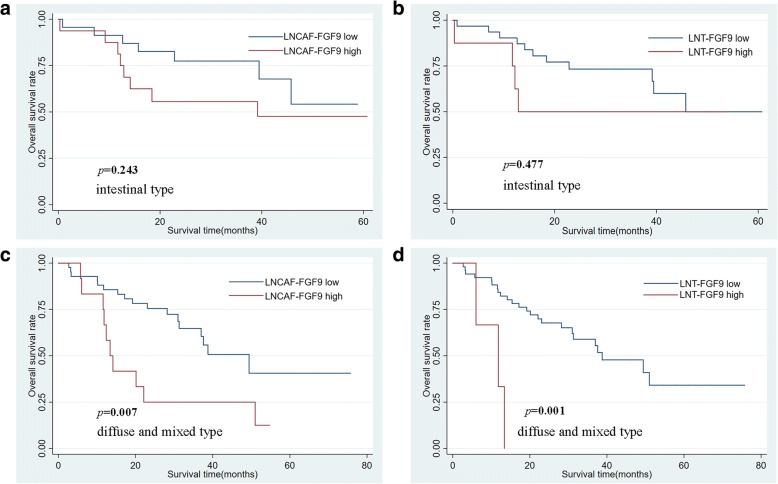
High FGF9 level in lymph node metastatic site CAFs and tumor cells were associated with poor prognosis in diffuse and mixed–type GC. **a** and **b** High FGF9 level in lymph node metastatic site CAFs or tumor cells were not associated with prognosis in intestinal-type GC. **c** and **d** High FGF9 level in lymph node metastatic CAFs or tumor cells was associated with poor prognosis in diffuse and mixed–type GC

Next, the relationship between different modes of FGF9 expression and clinicopathological parameters of gastric adenocarcinomas was analyzed (Table 1). In primary tumor CAFs, FGF9 expression status was only associated with tumor location (*P* < 0.05), while in primary tumor cells only associated with age (*P* < 0.05). What’s more, in lymph node metastatic site CAFs, the expression of FGF9 was related to tumor differentiation and Lauren type. The percentage of high FGF9 level in LNCAFs was substantially higher in intestinal-type GC compared with diffused and mixed–type GC (Fig. 5k, 45.6% vs 21.7%, *P* < 0.01). Likewise, the expression of FGF9 in lymph node metastatic site tumor cells was also connected with Lauren type. The proportion of high FGF9 level in LNT was much higher in intestinal-type GC than in diffused and mixed–type GC (Fig. 5l, 19.6% vs 6.7%, *P* < 0.05). The Kaplan–Meier survival analysis was performed to explore the relationship between FGF9 level and prognosis. Although the expression of FGF9 in PTCAFs, PT cells, and LNT cells was not associated with prognosis (Fig. 6a–c, *P* > 0.05), high FGF9 level in LNCAFs was closely associated with poor prognosis in patients with GC (Fig. 6 d, *P* < 0.05). Considering the discrepancy in the FGF9 level between different Lauren types, the Kaplan–Meier survival curves in intestinal-type GC and diffused and mixed–type GC were also assessed. In a word, though it wasn’t relevant with poor prognosis in LNCAFs or tumor cells of intestinal-type GC (Fig. 7a and b, *P* > 0.05), but the high FGF9 level was associated with dramatically poor prognosis in diffuse and mixed–type GC (Fig. 7c and d, *P* < 0.01).

### Expression of miR-214 was reduced in gastric cancer tissues

Expression of miR-214 was examined by ISH using 142 cases of gastric cancer tissues and matching normal gastric mucosa tissues. The expression of miR-214 was detected in the cytoplasm of cancer cells or normal gastric mucosa epithelial cells, but not in the CAFs and NFs (Additional file 2: Figure S1). Moreover, the expression of miR-214 was decreased in cancer cells compared to that in normal gastric mucosa epithelial cells (17.6% vs 41.5%, *P* < 0.01. Additional file 1: Table S3).

## Discussion

It is fully accepted that dysregulation of miRNAs in cancer cells are involved in cancer growth and progression [24–28], however, the function of miRNAs in CAFs of tumor microenvironment is ambiguous. As the most abundant stromal cells in cancer, CAFs play a key role in the communication with tumor cells and shape a supportive microenvironment for more aggressive behaviors of tumor cells [29]. Dysregulation of miRNAs in CAFs is increasingly recognized as boosters for tumor proliferation, invasion, and metastasis in many types of solid tumors such as breast cancer [30–32], ovarian cancer [16], and GC [13, 17, 18]. The present study showed that miRNA-214 expression significantly decreased in CAFs of GC compared with NFs. Furthermore, the upregulation of miRNA-214 in CAFs markedly inhibited the migration and invasion of GC cells, but failed to affect their proliferation. These findings suggested that anti-oncogenic miR-214 was responsible for the tumor-promoting ability of CAFs in GC. Actually, miRNA-214 acting as an oncogene or anti-oncogene in a certain type of cancer is still a controversial issue. Some researchers hold the view that miR-214 is a tumor suppressor. For example, the downregulation of miR-214 in intrahepatic cholangiocarcinoma promoted EMT by directly targeting the Twist gene [33]. And Shih et al. [34] found that decreased miR-214 level in human hepatocellular carcinoma (HCC) was associated with worse prognosis and contributed to de novo hypervascular HCC via induction of hepatoma-derived growth factor (HDGF) secretion [34]. On the contrary, miR-214 was regarded as an oncogene in other various tumors. Up-regulated miR-214 expression in breast cancer tissues markedly enhanced tumor cells invasion through suppressing p53 expression [35]. Similarly, miR-214 was overexpressed in GC and knockdown of miR-214 in GC cells significantly inhibited the proliferation, migration, and invasion capacity of cancer cells through targeting PTEN [36]. The disagreement in miR-214-driven aggressive behavior might be miR-214 regulated different genes via activating divergent predominant pathways in various cancer types. Nonetheless, these studies focused solely on tumor cells, without considering stromal cells within TME. Only one study demonstrated that downregulated expression of miR-214 in CAFs of ovarian cancer reprogrammed NFs to CAFs, which controlled invasion of cancer cells though stimulating the production and secretion of chemokine CCL5 into the TME. Yet, it is a necessary to understand the precise molecular mechanisms underlying this anti- or pro-tumor role of miR-214 [16].

It is known that EMT is a governing process in cancer metastasis. Dissemination of cells from primary tumor is facilitated by EMT, which allows epithelial-like tumor cells (cancer cells) to acquire invasive mesenchymal-like traits [37]. During cancer progression, a variety of stromal cell–derived signals synergize with one another to incite and maintain EMT in primary tumors, like paracrine signals generated by CAFs [38]. The epithelial cells undergoing EMT lose epithelial characteristics, such as loss of E-cadherin expression, and gain mesenchymal features, such as overexpression of Vimentin and N-cadherin. It has been realized that numerous transcription factors can induce EMT, including Snail, ZEB, and Twist. Previous research revealed that several miRNAs participated in modulating the tumorous EMT process. For example, the miR-200 family and miR-205 reported as the main miRNAs in influencing EMT were decreased in different types of tumor and boosted EMT progression via regulating the expression of ZEB family transcription factors [39]. MiR-214 was also deemed as an intrinsic modulator of EMT by targeting Twist1 in intrahepatic cholangiocarcinoma [33]. The majority of research finding about the association of miRNAs and EMT concentrated on cancer cells themselves, in which miRNAs regulate EMT by directly suppressing EMT related transcription factors. Nevertheless, there is a lack of data directed to the effect of CAFs-derived miRNAs on tumor EMT. Downregulation of miR-214 in gastric CAFs could accelerate the migration and invasion of GC cells by triggering the EMT process in current study. Specifically, a novel mechanism was proposed that CAFs-derived miRNA-214 through targeting FGF9 in itself to regulate the EMT event of tumor cells, highlighting the crosstalk between tumor cells and TME. Recently, it is showed that the exosomal delivery of miRNAs from CAFs into cancer cells resulted in the EMT of cancers. For instance, exosomal miR-148b was transferred from CAFs to endometrial cancer cells and then processed EMT of cancer cells by directly targeting DNMT1 [40]. Loss of exosomal miR-320a from CAFs mediated EMT in hepatocellular carcinoma cells by binding to its direct downstream target PBX3 [41]. That is to say, the definite impact of CAFs-derived exosomal miRNA-214 on adjacent cancer cells should be received more attentions in future work.

The growth factor FGF9 is a secretory protein belonging to the FGFs family. Several studies displayed that FGF9 is a key mediator of tumor progression in many human cancers, including lung adenocarcinoma, prostate cancer, and HCC [42–44]. Nevertheless, whether CAF-derived FGF9 was also involved in tumor progression was not delineated by these studies. Obviously, it is vital to stretch extended studies of the interaction between cancer cells and their surrounding stromal cells [45]. On the one hand, it is found that FGF9 was overexpressed in CAFs of GC compared with NFs. On the other, the effects of CAFs on the migration and invasion of GC cells could be significantly inhibited by adding FGF9-neutralizing antibody into the conditioning medium, suggesting the indispensable role of FGF9 in the tumor-promoting ability of CAFs in GC. Coincidently, Sun et al. [46] showed that CAF-derived FGF9 against apoptosis and enhances invasive capability of GC cells in vitro*,* but how did FGF9 work in CAFs? This study testified that the protein and mRNA expression of FGF9 was significantly decreased by miR-214 mimics in CAFs and miR-214 had a direct inhibitory effect on FGF9. Moreover, the dual luciferase reporter assay unmasked two binding sites between miR-214 and FGF9 3’UTR in favor of the forceful suppression effect of miR-214 on FGF9. In brief, these results indicated that miR-214 could inhibit the tumor-promoting ability of CAFs via directly targeting FGF9 in vitro.

Beyond that, the small tissue samples (20 cases) used in a previous study may not suffice to illuminate the real connection between FGF9 in gastric CAFs and clinicopathological features and prognosis [46]. Thus, in this study, immunohistochemistry (IHC) was performed on tissue microarrays harboring 160 primary GC tissues and corresponding 106 lymph node metastatic sites. The results showed that the FGF9 expression level was higher and more common in CAFs than that in tumor cells, which was present not only in primary tumors but also in lymph node metastatic sites, indicating that FGF9 was mainly secreted by CAFs rather than by cancer cells. Although the influence of FGF9 expression in tumor cells of primary tumor and lymph node metastatic sites on survival outcome was meaningless, high staining of FGF9 in CAFs of lymph node metastatic sites was indeed associated with poor prognosis. In other words, the high expression of FGF9 in gastric CAFs was more important than that in tumor cells themselves. Unlike in the lymph node metastatic sites, high FGF9 expression in CAFs in primary lesions had no effect on the prognosis, suggesting that FGF9 in CAFs had a tumor-promoting role principally in the advanced stage of tumor progression but not in the early stage. A rational explanation of its differences between primary and metastasis lesions is the heterogeneity of CAFs. Actually, CAFs have heterogeneous origins (resident-tissue derived fibroblasts, bone-marrow cells derived fibroblasts, vasculature system cells derived fibroblasts and cancer cells EMT), phenotypes (FAP, α-SMA, PDGFR-A, FSP-1), and functions (tumor-promoting or tumor-suppressive). The ultimate ending of CAFs impacting on tumor development may differ depending on the combination factors from cancer cells and the tumor stage [47]. Meanwhile, our study exhibited that different biological importance of CAFs may exist in primary tumor sites and lymph node metastases of GC. Of note, it was found that CAFs were involved in the pre-metastatic niche formation [48]. Therefore, compared CAFs from the primary tumor with that in metastases may better reflect the dynamic shift of TME during tumor metastasis and have more robust evidence to predict clinical process. In addition, one interesting fact that came out from the present study was the high FGF9 level in CAFs of lymph node metastatic sites was more commonly expressed in intestinal-type than diffused and mixed–type GC. In intestinal-type GC, the Kaplan–Meier survival analysis showed no correlation between FGF9 level in CAFs of lymph node metastatic sites and overall survival. On the contrary, the high FGF9 level in CAFs of lymph node metastatic sites showed inverse associations with good prognosis in diffused and mixed–type GC. The contradiction between the relationship of FGF9 expression and prognosis in different Lauren type might be interpreted as distinct molecular properties and tumor progression between intestinal- and diffuse-type gastric adenocarcinoma. In summary, the pro-tumor activities of FGF9 in tumor progression was more pronounced in diffused and mixed–type GC than in intestinal-type GC, despite the high expression of FGF9 in CAFs of intestinal-type GC. Therefore, further studies based on CAFs from different Lauren type of GC will be helpful to design valuable treatment strategies.

It has recently demonstrated that combined therapeutic approach targeting both cancer cells and TME angiogenesis could decrease tumor growth and tumor metastasis in a (patient-derived xenograft) PDX model of colorectal cancer [49]. For this reason, a real feasible and effective treatment option is ought to target not only on tumor cells themselves, but also on TME. The latest therapeutic strategy that aimed at eliminating CAFs or reverting the activated state of CAFs back to quiescence may pave the way for new therapeutic opportunities, however, there are still many challenges ahead and areas that need improvement for this therapy [50]. Finally, the results obtained from this study may provide an insight into the choice of candidate target for combination therapy targeting the crosstalk between cancer cells and CAFs.

## Conclusions

In conclusion, the study revealed that miR-214, as a novel tumor suppressor gene, was downregulated in CAFs of GC. And miR-214 was critical to the tumor-promoting effects of CAFs on the migration and invasion of GC cells through suppressing the EMT process. Besides, miRNA-214 in CAFs directly regulated FGF9 expression. FGF9 could promote the migration and invasion ability of GC cells in vitro. Moreover, FGF9 high expression in CAFs of lymph node metastatic sites was associated with poor prognosis in GC. Consequently, the intervention of miR-214/FGF9 axis in CAFs would be considered as an effective therapeutic target for GC.

## Additional files

Additional file 1: **Table S1.** Sequence of miR-214 mimic and negative control. **Table S2.** Primers used in Real time-PCR test. **Table S3.** Expression of miR-214 in gastric cancer tissue. (DOCX 18 kb)

Additional file 2: **Figure S1.** Expression of miR-214 in gastric cancer tissues. (a) The expression of miR-214 is strong positive in normal gastric mucosa epithelial cells (★), but negative in NFs (▲). (b) The expression of miR-214 is weakly positive in gastric cancer cells (★), but negative in CAFs(▲). (DOCX 18 kb) (TIF 6161 kb)

## Abbreviations

3’UTR: 3′-Untranslated region.
CAFs: Cancer-associated fibroblasts
CCL4: C-C motif chemokine ligand 4
CCL5: C-C motif chemokine ligand 5
CM: Conditioned medium
CSF1: Colony-stimulating factor 1
CXCL14: C-X-C motif chemokine ligand 14
EMT: Epithelial–mesenchymal transition
FAP: Fibroblast activation protein
FGF: Fibroblast growth factor
GC: Gastric cancer
HCC: Hepatocellular carcinoma
HDGF: Hepatoma-derived growth factor
IHC: Immunohistochemistry
IL3: Interleukin 3
LN: Lymph node
LNT: Lymph node metastatic site tumor cells
NFs: Normal fibroblasts
PT: Primary tumor
TME: Tumor microenvironment
