# Supplementary material for: Downregulation of miRNA-214 in cancer-associated fibroblasts contributes to migration and invasion of gastric cancer cells through targeting FGF9 and inducing EMT
Source: J Exp Clin Cancer Res. 2019 Jan 15;38:20. doi: 10.1186/s13046-018-0995-9 (PMC6334467; doi:10.1186/s13046-018-0995-9)
Supplement: Supplementary file 1 — Table S1. Sequence of miR-214 mimic and negative control. Table S2. Primers used in Real time-PCR test. Table S3. Expression of miR-214 in gastric cancer tissue. (DOCX 18 kb) [file 13046_2018_995_MOESM1_ESM.docx]

Supplementary Table 1 Sequence of miR-214 mimic and negative control

| Oligonucleotides | sense(5'-3') | antisence(5'-3') |
| --- | --- | --- |
| miR-214 mimic | ACAGCAGGCACAGACAGGCAGU | UGCCUGUCUGUGCCUGCUGUUU |
| miR-NC | UUCUCCGAACGUGUCACGUTT | ACUUGACACGUUCGGAGAATT |

Supplementary Table 2 Primers used in Real time-PCR test

| Primer | Forward(5'-3') | Reverse(5'-3') |
| --- | --- | --- |
| FAP | AATGAGAGCACTCACACTGAAG | CCGATCAGGTGATAAGCCGTAAT |
| FGF9 | GGGGAGCTGTATGGATCAGA | GTGAATTTCTGGTGCCGTTT |
| IL3 | CCTTTGCCTTTGCTGGACTTC | TTGACAGCCCTGTTGAATGC |
| FGF14 | GTTGCCATGTACCGAGAACC | GTTGACTGGTTTGCCTCCAT |
| CSF1 | TGCGTCCGAACTTTCTATG | CACTGCTAGGGATGGCTTT |
| FGF11 | TCTCTCTCCAGAGCCTCAGC | TGGGTGAAGGAGCTGGTATC |
| FGF1 | GGCTCACAGACACCAAATGA | CTTGAGGCCAACAAACCAAT |
| FGF7 | GACATGGATCCTGCCAACTT | GCCATTTGCTCTGGAGTCAT |
| CCL4 | CTTCCTCGCAACTTTGTGGT | CCAGGATTCACTGGGATCAG |
| FGF10 | GCGGAGCTACAATCACCTTC | TGTACGGGCAGTTCTCCTTC |
| CXCL14 | CACTGCGAGGAGAAGATGGT | GGCGTTGTACCACTTGATGA |
| GAPDH | GTGAAGGTCGGAGTCAACG | TGAGGTCAATGAAGGGGTC |
| miR-214 | TACAGCAGGCACAGACAGGCA |  |

Supplementary Table 3 Expression of miR-214 in gastric cancer tissue

| Groups | Positive (%) | Negative (%) | Total (n) | *P* value |
| --- | --- | --- | --- | --- |
| Normal tissues | 25(17.6%) | 117(82.4%) | 142 | 0.000 |
| Cancer tissues | 59(41.5%) | 83(58.5%) | 142 |  |
