# Supplementary figures and images for: Downregulation of miRNA-214 in cancer-associated fibroblasts contributes to migration and invasion of gastric cancer cells through targeting FGF9 and inducing EMT
Source: J Exp Clin Cancer Res. 2019 Jan 15;38:20. doi: 10.1186/s13046-018-0995-9 (PMC6334467; doi:10.1186/s13046-018-0995-9)

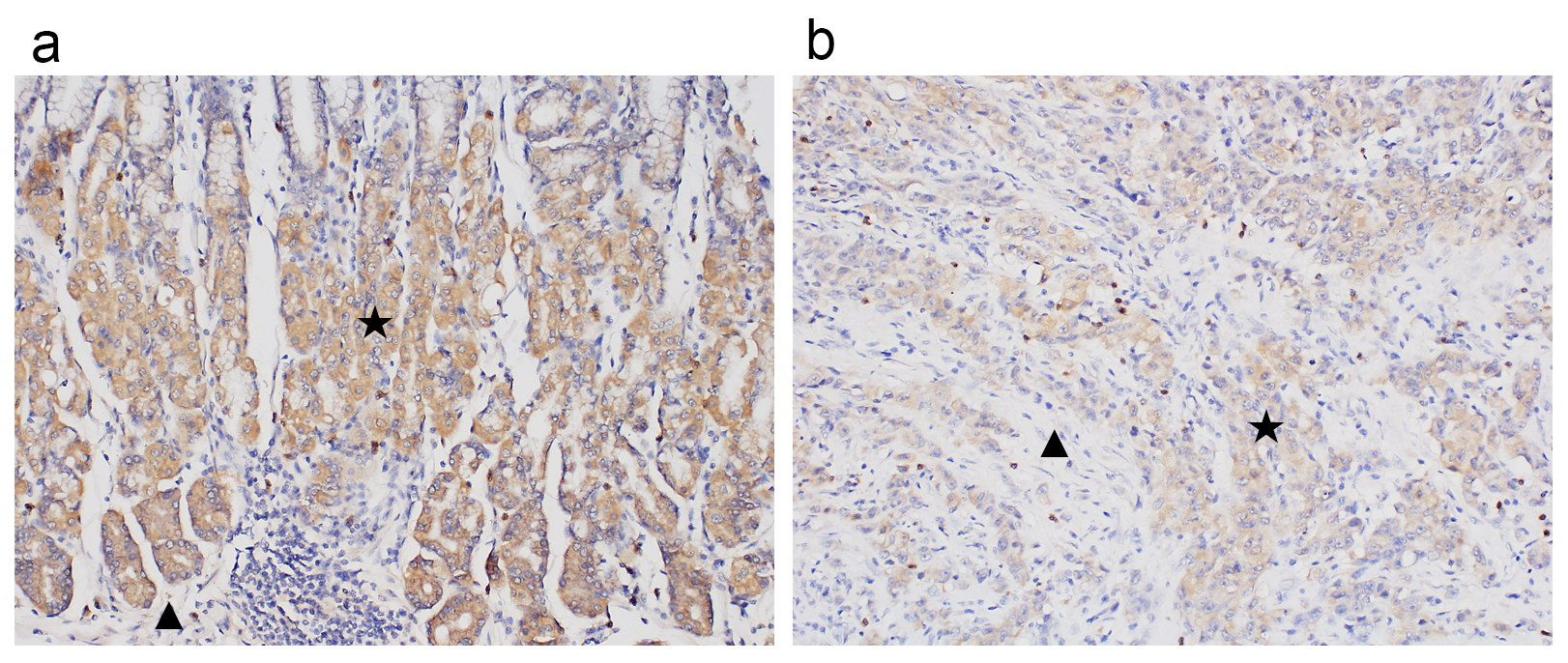

Supplement: Supplementary file 2 — Figure S1. Expression of miR-214 in gastric cancer tissues. (a) The expression of miR-214 is strong positive in normal gastric mucosa epithelial cells (★), but negative in NFs (▲). (b) The expression of miR-214 is weakly positive in gastric cancer cells (★), but negative in CAFs(▲). (DOCX 18 kb) (TIF 6161 kb) [file 13046_2018_995_MOESM2_ESM.tif]
